# Design of a Remote Coaching Program to Bridge the Gap From Hospital Discharge to Cardiac Rehabilitation: Intervention Mapping Study

**DOI:** 10.2196/34974

**Published:** 2022-05-25

**Authors:** Paul Keessen, Ingrid CD van Duijvenbode, Corine HM Latour, Roderik A Kraaijenhagen, Veronica R Janssen, Harald T Jørstad, Wilma JM Scholte op Reimer, Bart Visser

**Affiliations:** 1 Centre of Expertise Urban Vitality, Faculty of Health Amsterdam University of Applied Sciences Amsterdam Netherlands; 2 Cardiovitaal Cardiac Rehabilitation Amsterdam Netherlands; 3 Department of Cardiology Leiden University Medical Center Leiden Netherlands; 4 Department of Cardiology, Heart Center Amsterdam University Medical Center Amsterdam Netherlands

**Keywords:** coronary artery disease, intervention mapping approach, information needs, support needs, eHealth, cardiac rehabilitation, remote coaching, e-coaching

## Abstract

**Background:**

Remote coaching might be suited for providing information and support to patients with coronary artery disease (CAD) in the vulnerable phase between hospital discharge and the start of cardiac rehabilitation (CR).

**Objective:**

The goal of the research was to explore and summarize information and support needs of patients with CAD and develop an early remote coaching program providing tailored information and support.

**Methods:**

We used the intervention mapping approach to develop a remote coaching program. Three steps were completed in this study: (1) identification of information and support needs in patients with CAD, using an exploratory literature study and semistructured interviews, (2) definition of program objectives, and (3) selection of theory-based methods and practical intervention strategies.

**Results:**

Our exploratory literature study (n=38) and semistructured interviews (n=17) identified that after hospital discharge, patients with CAD report a need for tailored information and support about CAD itself and the specific treatment procedures, medication and side effects, physical activity, and psychological distress. Based on the preceding steps, we defined the following program objectives: (1) patients gain knowledge on how CAD and revascularization affect their bodies and health, (2) patients gain knowledge about medication and side effects and adhere to their treatment plan, (3) patients know which daily physical activities they can and can’t do safely after hospital discharge and are physically active, and (4) patients know the psychosocial consequences of CAD and know how to discriminate between harmful and harmless body signals. Based on the preceding steps, a remote coaching program was developed with the theory of health behavior change as a theoretical framework with behavioral counseling and video modeling as practical strategies for the program.

**Conclusions:**

This study shows that after (acute) cardiac hospitalization, patients are in need of information and support about CAD and revascularization, medication and side effects, physical activity, and psychological distress. In this study, we present the design of an early remote coaching program based on the needs of patients with CAD. The development of this program constitutes a step in the process of bridging the gap from hospital discharge to start of CR.

## Introduction

Cardiac rehabilitation (CR) is a cornerstone of secondary prevention and has been shown to reduce cardiovascular mortality and hospital readmissions and improve psychological well-being [1,2]. Although early enrollment in CR is advised, patients with coronary artery disease (CAD) generally wait 4 to 6 weeks after hospital discharge before starting physical CR [3,4]. This waiting period constitutes a gap between hospital discharge and the start of CR. Since patients are often discharged within 2 to 4 days, there is little room for patient education while patients are often in need of tailored medical information and support [5]. In addition, symptoms of anxiety are present in 28% and depression in 18% of patients entering CR, which negatively impacts adherence to CR [6]. While patients are sometimes offered educational support programs after hospitalization and prior to the start of CR that have been shown to increase knowledge and promote health behavior, these interventions are frequently neither initiated nor adhered to [7,8].

A potentially promising strategy for provision of information and support directly after hospital discharge is the use of a remote coaching program. In this study, remote coaching is defined as an online communication system used to provide medical, psychological, and social support to patients at home. Remote coaching programs, as part of a CR program, improve patients’ physical capacity, clinical status, and psychosocial health [9]. Moreover, remote coaching has the potential of improving self-efficacy, which in turn is associated with improved CR adherence [10-12].

Nevertheless, it is unknown whether remote coaching meets patients’ needs in the early phase (ie, gap) after hospital discharge. If the specific information and support needs in the early phase after hospital discharge are known, a remote coaching program to bridge the gap from hospital discharge to the start of CR can be developed.

Therefore, the objective of this study was to explore the information and support needs directly after hospital discharge among patients with CAD and develop an early remote coaching program to provide tailored information and support.

## Methods

### Study Design

In this study, we used the intervention mapping (IM) approach, a systematic and comprehensive methodology grounded in theory and developed in collaboration with key stakeholders (health care providers, patients, and informal caregivers) [13,14]. The 6 steps of the IM approach are (1) identification of the problem by performing a needs assessment, (2) identification of outcomes and change objectives, (3) selection of theory-based intervention methods and practical strategies, (4) development of an intervention, (5) generation of an adoption and implementation plan, and (6) generation of an evaluation plan. In this study, we completed the first 3 steps of the IM protocol.

The development of this remote intervention was performed on the existing platform of Cardiovitaal Cardiac Rehabilitation Amsterdam currently only used by patients who have started outpatient physical rehabilitation. This existing digital platform is used by patients to monitor physical (eg, blood pressure and steps per day) and psychological (symptoms of anxiety and depression) health. In addition, patients and health care providers use this platform to communicate using videocalling or the chat function. The design of the early coaching program was incorporated in the existing digital platform. At the beginning of this study, a multidisciplinary research group of health care providers comprised physical therapists (PK, ICDvD), physical therapist (Ilonka Pol), psychologist (VRJ), cardiologist (RAK), and registered nurse (Christine Dolman). All participants had expertise in the field of CR. The research group met on 3 occasions to discuss each step. The authors (PK and ICDvD) completed each step only after group consensus was reached. During each step the following tasks were completed.

#### Step 1. Logic Model of the Problem

The overall objective of step 1 was to define a logic model of the problem. The information and support needs of patients with CAD were investigated with an exploratory literature review and semistructured interviews with key stakeholders.

On the basis of these findings, a logic model of the problem was created using the Predisposing, Reinforcing, and Enabling Constructs in Educational Diagnosis and Evaluation (PRECEDE) model, which is used as framework to identify intervention strategies [15]. After completion of the model, program objectives and outcomes were formulated by the research group. Based on these findings, outcomes and change objectives were formulated in step 2.

#### Step 2. Logic Model of Change

The overall objective of step 2 was to define a logic model of change. First, expected outcomes were formulated based on the results of step 1. These outcomes concern behavioral outcomes (outcomes related to the patient level) and environmental outcomes (outcomes related to the social network of the patient and health care setting). Second, based on the outcomes, we formulated program objectives. In the IM approach, these are formulated as performance objectives (outcomes that describe the desired behavior). Performance objectives were formulated at the patient and environmental level. Third, the research group selected determinants to influence during the intervention and created matrices of change objectives. Finally, a logic model of change was created. The logic model of change and matrices of change form the basis of this intervention and were further elaborated on in step 3 where they were matched with theory-based intervention methods and practical strategies.

#### Step 3. Program Design

The overall objective of step 3 was to select theory-based intervention methods and practical strategies as ingredients for an intervention. As starting point for IM step 4, a preliminary design of an intervention was developed. Consecutive tasks were completed by the research group. First, based on the preceding steps, the overall themes, components, and sequence of the intervention were determined. Second, theory- and evidence-based change methods were selected and matched with the overall themes and components of the intervention. Third, the research group discussed and selected practical applications to deliver the intervention. Last, the design of the intervention was presented.

### Study Population

For this study, patients were identified through an electronic patient file search of the Amsterdam University Medical Center and approached by telephone shortly after hospital discharge (2 to 4 days). We aimed to include a heterogenous sample to obtain a wide variety of viewpoints to increase the generalizability of our program.

We included patients with an acute coronary syndrome (ACS), coronary revascularization (percutaneous coronary intervention [PCI] or coronary artery bypass grafting [CABG]), or referral to CR. Patients were excluded if they had cognitive problems (Mini-Mental State Exam <24) or were unable to speak Dutch. Recruitment of patients ended when no new information was discovered in the data analysis (data saturation).

### Ethics Approval

The Medical Ethics Committee of the Amsterdam University Medical Center approved the study protocol (NL65218.018.18).

### Exploratory Literature Study

An exploratory literature review synthesizes the extant literature and usually identifies the gaps in knowledge that an empirical study addresses [16]. The objective of our exploratory literature review was to assess information and support needs after (acute) cardiac hospitalization. A comprehensive search was performed in PubMed to identify relevant studies concerning this topic. For this search, automatic term mapping was used to match the entered terms with Medical Subject Headings to enhance the search strategy. The following terms were used in the search builder: (coronary artery disease) OR (acute coronary syndrome) OR (percutaneous coronary intervention) OR (coronary artery bypass graft) AND (information needs OR support needs). The search strategy included terms occurring in the title and main text with no restrictions for date range but limited to the English language. During the screening process, articles were selected on title, abstract, and full text. The literature search was conducted by ICDvD and continuously discussed with PK and BV.

### Semistructured Interviews

Semistructured interviews were conducted to assess patients’ information and support needs. An interview guide was developed by the research group, in several rounds, until consensus was reached about the final version (Multimedia Appendix 1). These 30-minute interviews took place at the Amsterdam University Medical Center or at the patient’s home. During the COVID-19 pandemic, interviews were continued by telephone. All patients gave informed consent for their personal data being used in this study. Interviews were performed by 2 physical therapists (PK and ICDvD), a physician assistant (Tarik Hoek Spaans), and 2 registered nurses (Bonita Meek and Miranda Balfoort).

All interviews were transcribed and were analyzed with qualitative data analysis software (MAXQDA 2018, VERBI GmbH). Three types of coding were used consecutively: open, axial, and selective. Initial codes were created by studying the segmented information. The codes were then abstracted into categories and subcategories. The underlying meanings of these categories were linked together to create overall themes. All data were independently coded for themes by 2 researchers (PK, ICDvD). A third researcher (BV) reviewed all codes and decided appropriate themes together with PK and ICDvD.

## Results

### Step 1. Logic Model of the Problem

**Figure 1:**
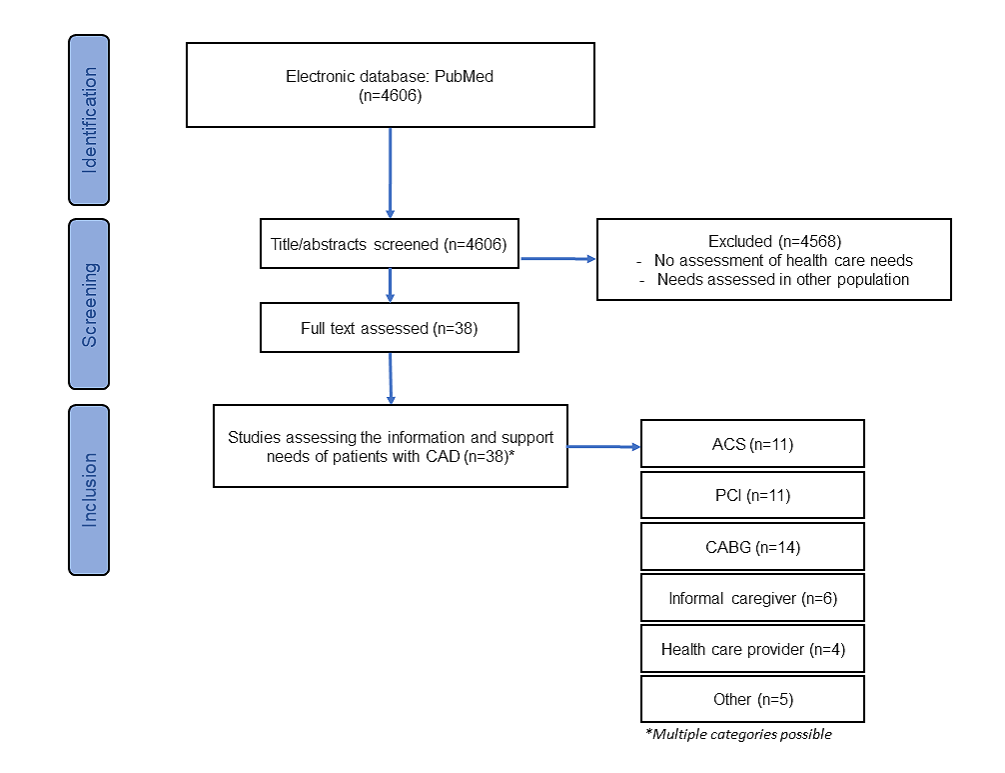
Flowchart of study. ACS: acute coronary syndrome; CABG: coronary artery bypass grafting; CAD: coronary artery disease; PCI: percutaneous coronary intervention.

The initial exploratory literature search identified 4606 electronic database papers. After removal of duplicates and non-English articles, the remaining papers were screened by title and abstract. After the screening procedure (see Figure 1 for flowchart), 38 articles were studied to identify the information and support needs of cardiac patients (see Multimedia Appendix 2 for an extraction table of our literature search).

Patients report a lack of (consistent) information after hospital discharge and that information needs were not always correctly perceived by health care providers [17-19]. The highest priority of information needs comprised information about medication and side effects, wound care, postoperative pain, physical activity, and dealing with emotions [20-28].

The greatest needs of information were found in young and middle-aged patients with a higher education [20,21]. No differences in type of information needs were found between men and women; however, women preferred to receive information before revascularization while men preferred it afterward [29,30]. Patients who were hospitalized after an acute coronary event were in greater need of information and emotional support than those treated electively [31].

High levels of anxiety were reported in the weeks after hospital discharge, especially in female patients and those with a lower education [32,33]. Patients reported distressing body signals, difficulties with sleeping, and insecurity about engaging in physical activity and returning to work [20,32,34-36]. Spouses reported high levels of psychological distress linked to anxiety, financial worry, and loneliness [37-39], highlighting a need to include spouses and informal caregivers in decision making and support programs [40-42].

In general, patient knowledge about risk factors and management of heart disease was limited, and patients often attributed the cause of their disease to nonmodifiable risk factors (ie, age, heredity) instead of lifestyle factors such as smoking, lack of physical activity, and unhealthy food choices [43-46]. However, reduction of mortality risk was rated as most important by patients with ACS [47].

In addition to having difficulties with understanding medical information, patients experienced problems with the referral process from hospital discharge to CR, which in turn led to a discontinuity in the health care process [48]. Those with a lower socioeconomic status felt especially excluded from CR while also having high information needs [49]. Moreover, these patients tended to have health beliefs that were not based on medical evidence, a predictor of nonadherence to CR [50,51]. Patients described advanced communication skills and pedagogical competences as important skills for health care providers [52]. Furthermore, the ability to build trust and tailor information to the individual were described by patients as important skills for health care providers [53,54].

### Interviews

Data saturation was achieved after 17 eligible patients were included. Ten patients participated in an interview at the hospital or at home, and 7 patients took part in telephone interviews. Our total study population comprised 17 patients (9 females) with a median age of 64 years. Ten patients were diagnosed with ACS and 7 patients with angina pectoris. An overview of baseline characteristics is presented in Table 1.

Our data revealed 6 main themes: psychological distress, distressing body signals, lack of information at hospital discharge, passive coping style, disrupted health care process after hospital discharge, and social support. Qualitative findings with reference to individual patients can be found in Table 1.

**Table 1:** Baseline characteristics.

Patient	Sex	Age range (years)	Diagnosis/intervention	Cardiac disease history	Comorbidity
1	Male	60-69	NSTEMI^a^/PCI^b^	Stroke, hypertension	HIV
2	Female	70-79	STEMI^c^/PCI	Hypertension, hypercholesterolemia	Hypothyroid
3	Male	80-89	NSTEMI/PCI	AF^d^	—^e^
4	Male	70-79	NSTEMI/PCI	Hypertension, hypercholesterolemia	Urothelial carcinoma
5	Male	50-59	STEMI/PCI	Hypertension, hypercholesterolemia	—
6	Male	60-69	STEMI/PCI	—	—
7	Female	70-79	AP^f^/PCI	Stroke	Hypothyroid, cholelithiasis
8	Female	50-59	STEMI/PCI	—	—
9	Male	60-69	NSTEMI/PCI	Diabetes mellitus, hypertension, OSAS^g^	Respiratory infection
10	Female	50-59	STEMI/PCI	Hypertension, hyperglycemia	—
11	Male	60-69	AP/CABG^h^	Stroke, hypertension	Rheumatic disease
12	Male	60-69	AP/CABG	—	—
13	Female	50-59	NSTEMI/PCI	Hypertension, hypercholesterolemia	Asthma, lumbar radicular syndrome
14	Female	50-59	AP/CABG	Stroke	Obesity
15	Female	60-69	AP/CABG	Complications during PCI, AF	—
16	Male	50-59	STEMI/PCI	—	Diabetes
17	Female	50-59	STEMI/PCI	—	Mixed connective tissue disease

^a^NSTEMI: non–ST-elevation myocardial infarction.

^b^PCI: percutaneous coronary intervention.

^c^STEMI: ST-elevation myocardial infarction.

^d^AF: atrial fibrillation.

^e^Not applicable.

^f^AP: angina pectoris.

^g^OSAS: obstructive sleep apnea syndrome.

^h^CABG: coronary artery bypass grafting.

#### Psychological Distress and Distressing Body Signals

After hospital discharge, patients reported having symptoms of anxiety and depression. In addition, patients reported needing information and support on dealing with body signals such as fatigue, palpitations, and wound pain after thoracotomy or PCI. These body signals often made patients afraid to engage in physical activity, which in some cases led to fear of bodily sensations and patients monitoring their heartbeat. See Multimedia Appendix 3 for the complete list of quotations.

I don’t want to feel that pain anymore.

P11, subcategory: wound pain/chest pain

I don’t dare to do anything.

P16, subcategory: hypervigilance

I monitor my heart rate.

P17, subcategory: fear of bodily sensations

Nothing will be the same again.

P16, subcategory: depression

#### Lack of (Consistent) Information at Hospital Discharge

Patients reported a lack of information at hospital discharge or stated that the information was vague or inconsistent. A major concern for patients was a lack of information about side effects of medication, which in some cases led to misinterpretation of body signals and revisiting the emergency room.

Patients also reported that they did not know the amount of physical activity they were allowed to engage in after hospital discharge, resulting in reluctance to engage in any physical activity whatsoever. Patients reported needing to be reassured about the chance of a new cardiac event before hospital discharge.

Not being reassured by a treating physician led, in some cases, to false beliefs about the procedure (eg, one patient believed a stent could shift in the artery by doing physical activity).

I felt a weird pressure on my chest, like my heart skipped a beat. I panicked, so I went back to the emergency room where they examined me. Afterward they told me it was a side effect of metropolol.

P10, subcategory: side effects medication

I don’t know if I can do any physical activity and if I injure my body if do physical activity.

P4, subcategory: physical activity

One health care provider tells me this, the other tells me that.

P1, subcategory: inconsistent information

I really missed talking to my physician about what had happened to my heart before I left the hospital.

P7, subcategory: cardiac event

#### Passive Coping Style

After hospitalization, patients often developed a passive coping style by spending all their time on the couch or in bed. In several cases, the informal caregiver performed all household chores and therefore felt overloaded. This maladaptive coping strategy was attributed to psychological distress and the inability to cope with distressing body signals.

I’d rather be in bed all the time.

P16, subcategory: inactivity

I did not do anything for 6 weeks, I’m just staying in bed and on the couch, I can’t do much more.

P9, subcategory: inactivity

#### Disrupted Health Care Process After Hospital Discharge

Patients reported problems with continuity of care, especially about the long interval between hospital discharge and the start of CR. For some patients, the relevance of CR was unclear, which made them reluctant to participate in CR.

I think the time between discharge and CR is too long.

P12, subcategory: time until CR

What is there to rehabilitate about the heart?

P2, subcategory: relevance of CR

The referral to CR went completely wrong. It took ages before it was clear where I needed to go and what was expected. Thinking about this makes me short of breath again.

P4, subcategory: negative experience hospital

#### Social Support

In the phase after cardiac hospitalization, patients received support from their informal caregivers. In some cases, the caregiver was overprotective and took all physical tasks out of the hands of the patient. Although well intentioned, this has a negative effect, since it is necessary that the patient undertakes activities for optimal recovery.

During the interviews, patients expressed their support needs and stated that receiving guidance and support, especially during physical activity or exercise, was paramount in regaining confidence.

If I do too much and I have complaints, my husband becomes angry and tells me to sit down.

P10, subcategory: hypervigilance informal caregiver

My husband does all the groceries and cooking and tells me to relax.

P7, subcategory: hypervigilance informal caregiver

I want to participate in CR to gain confidence so that afterward I can start exercising alone.

P5, subcategory: CR

I would feel anxious if I started exercising without guidance. It’s about confidence. I can do it, but it would not feel right.

P16, subcategory: CR

**Figure 2:**
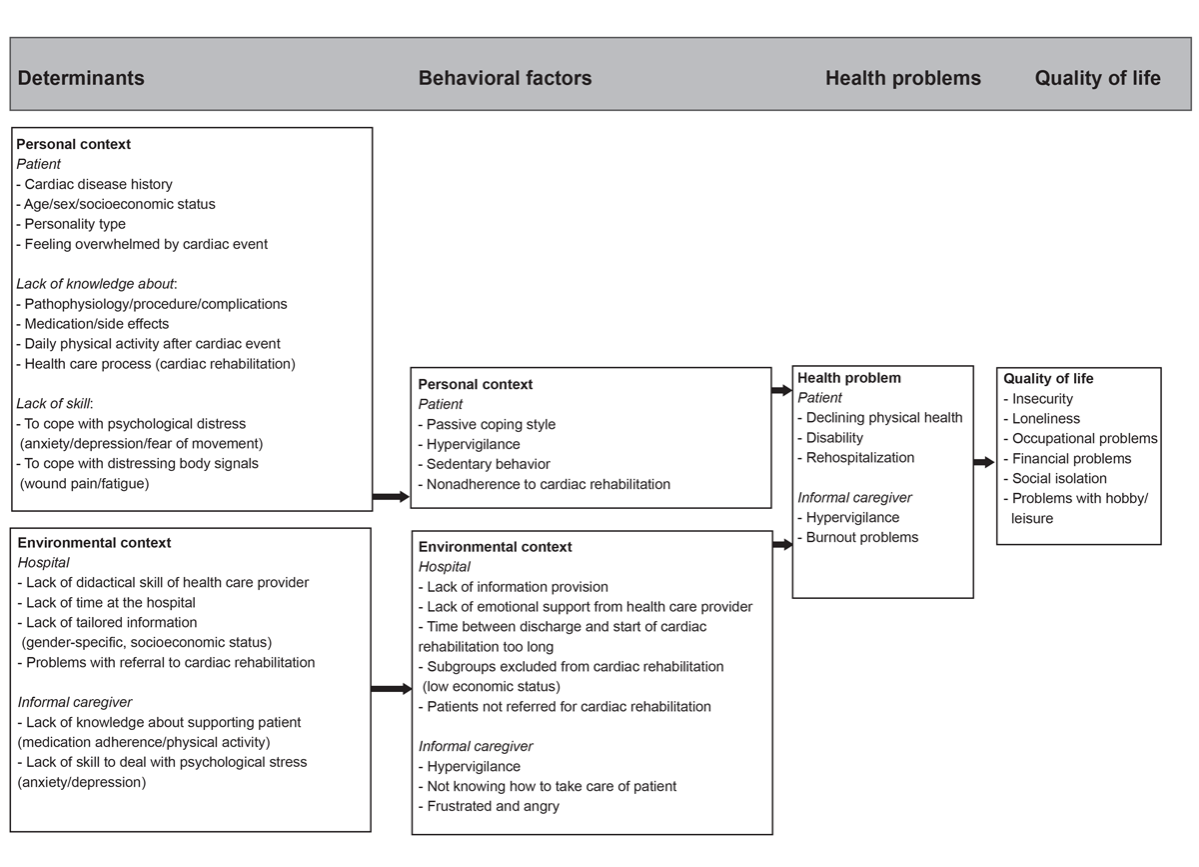
Logic model of the problem.

Findings from the exploratory literature study and interviews were divided into the categories determinants, behavioral factors, health problems, and quality of life and compiled in the PRECEDE-based logic model of the problem by PK and IvD. After the research group discussed the model and proposed several adjustments, the final model was developed. The final model is presented in Figure 2.

Based on the logic model of the problem, the overall goal was determined by the research group. The overall goal of this intervention is to bridge the gap from hospital discharge to CR by stimulating self-management behavior and providing tailored illness management information and psychological support to patients and their informal caregivers by means of a remote coaching program.

### Step 2. Logic Model of Change

The logic model of change was developed based on the findings in step 1. First, behavioral and environmental outcomes were defined. Second, the influence of these outcomes on the health problem and quality of life was described. Third, performance objectives were formulated for the behavioral and environmental outcomes. Fourth, a theoretical framework and determinants to influence were selected. Last, a determinant matrix was developed that describes how each determinant is related to the performance objectives.

#### Behavioral Outcomes

Patients and informal caregivers actively prevent physical and psychological problems by adhering to a remote CR program in the first phase after hospital discharge.

#### Environmental Outcomes

The CR center supports patients and informal caregivers in the first 3 weeks after hospital discharge by providing tailored information and (emotional) support.

### Program Objectives

#### Behavioral and Environmental Outcomes

For the behavioral outcomes, 4 performance objectives were formulated:

- Patients and informal caregivers gain knowledge on how CAD and revascularization affects their bodies and health
- Patients and informal caregivers gain knowledge about medications and side effects and adhere to their treatment plan
- Patients and informal caregivers know which daily physical activities they can and can’t do safely after hospital discharge and are physically active
- Patients and informal caregivers can deal with the psychosocial consequences of CAD

For the environmental outcomes, 2 performance objectives were formulated:

- In the 3 weeks after hospital discharge, patients and informal caregivers needs are assessed by the health care professional
- Health care providers give tailored information and coach cardiac patients and their informal caregivers in the first phase after hospital discharge

#### Theoretical Framework

**Figure 3:**
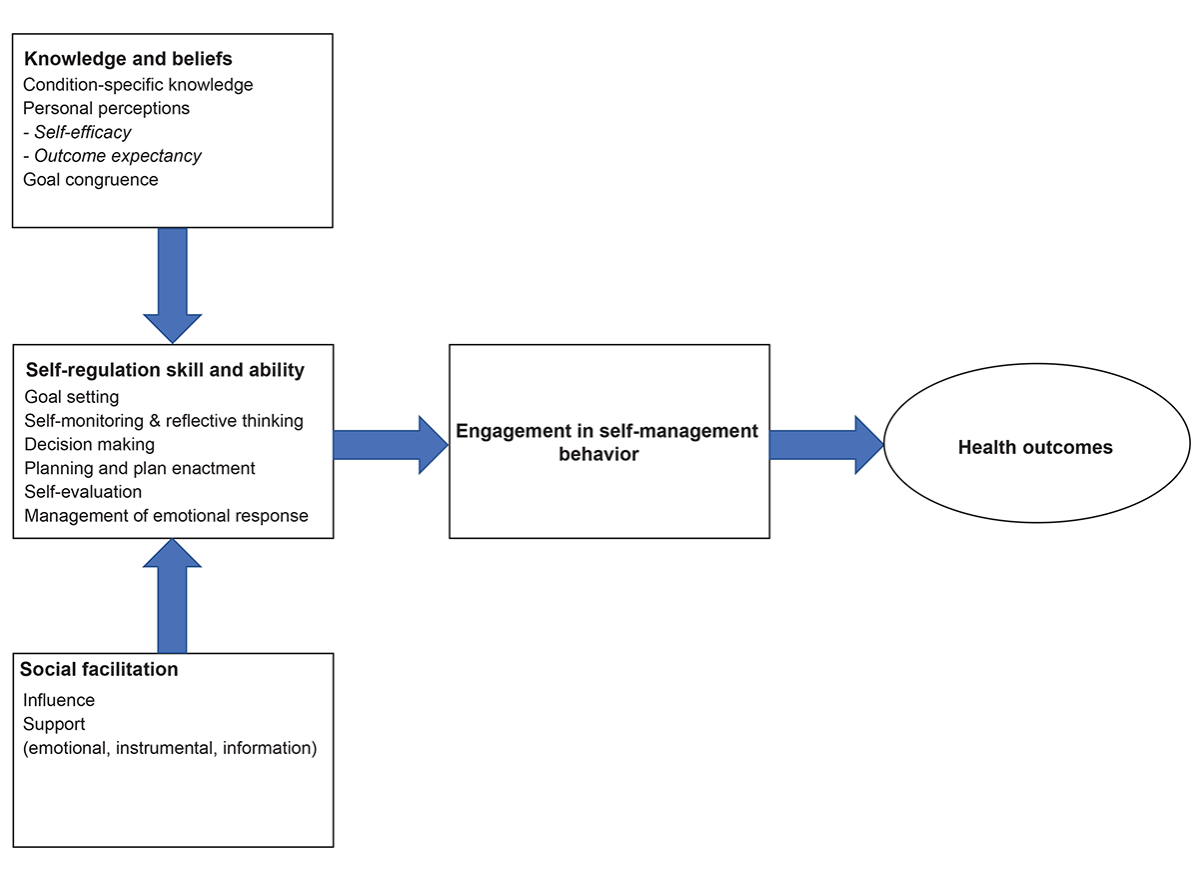
Theory of health behavior change.

The research group chose the theory of health behavior change (THBC) and theory of planned behavior (TPB) as the theoretical framework for this intervention. The THBC is presented in Figure 3.

According to THBC, 3 main determinants influence the adoption of self-management behavior [55]. These determinants are knowledge, self-regulatory skills, and abilities and social facilitation [55]. The TPB links beliefs to behavior and states that an individual’s behavioral intentions are shaped by attitude, subjective norms, and perceived behavioral control [56]. According to these theories, knowledge is defined as part of an attitude toward a certain behavior, which in turn is based on personality traits, values, preference, and outcome expectancy. Self-regulatory skills refer to the process of incorporating behavior change in daily life [56]. In this study, self-regulatory skills can be described as patients monitoring themselves (eg, body signals, emotions), goal setting (eg, performing daily physical activity), reflective thinking (eg, effects of cardiac event of health and quality of life), planning (eg, medication adherence, appointments with physician), decision making (eg, lifestyle habits), plan enactment (eg, setting feasible goals), self-evaluation, and management of emotions arising as a result of behavior (eg, feeling anxious or depressed). Social facilitation is divided in social influence and social support and refers to the health care provider providing credible information and social support to the patient and informal caregiver.

Based on the core components of the THBC and TPB, the research group chose the following determinants of influence for the remote coaching program: knowledge, skills, attitude, social influence, and self-efficacy. These determinants were used to create a matrix where all performance objectives were described per determinant. A detailed description of all determinants is presented in Multimedia Appendix 4.

**Figure 4:**
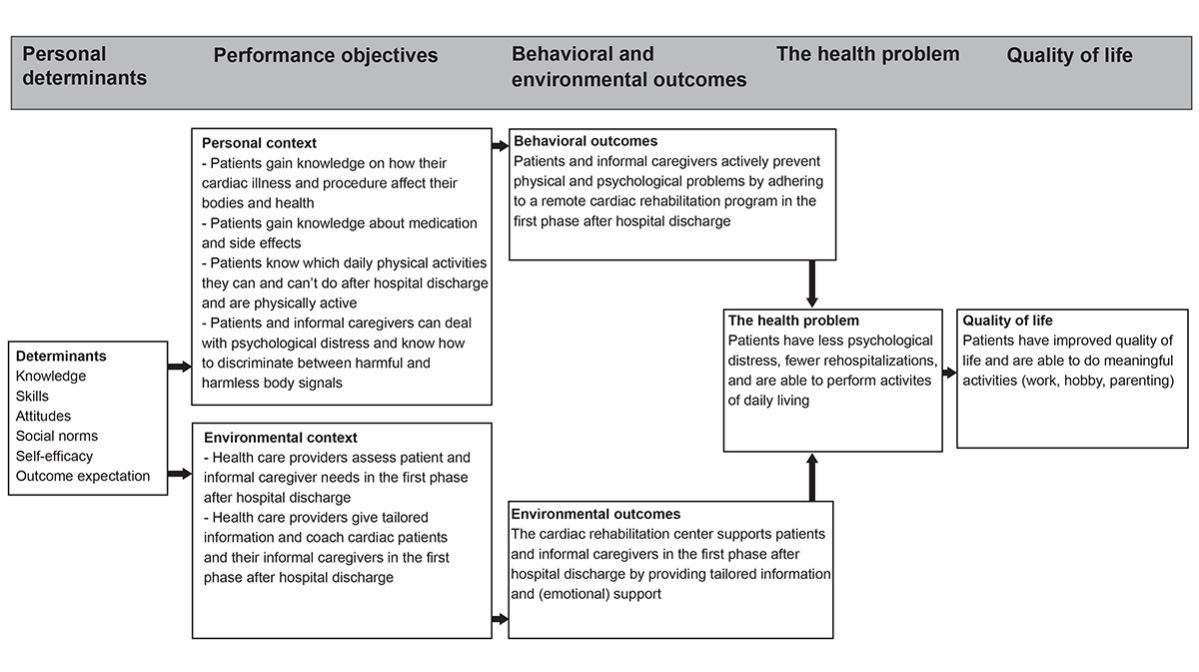
Logic model of change.

The last task of step 2 was to create a model of change which represents the relationship between the determinants, performance objectives, and desired outcomes (Figure 4).

### Step 3. Program Design

The results from step 2 were used to design the program. In this step, the research group matched the 6 determinants and performance objectives with theory-based and practical strategies, in line with the IM taxonomy [57]. The selection of theory-based strategies was based on the theoretical framework of Kok et al [57]; please see this paper for a detailed explanation of the theory-based strategies.

#### Selection of Theory-Based and Practical Strategies

Before specifically discussing the program objectives in relation to their determinants, the research group freely discussed the program design of this intervention based on the findings in step 1 and 2. The research group agreed that a remote coaching program to bridge the gap between hospital discharge and the start of CR was relevant. The research group defined 2 core elements of the program: behavioral counseling and increasing knowledge by using health video clips.

#### Behavioral Counseling

Health care providers will contact patients and informal caregivers within 2 days after hospital discharge using an eHealth platform. The use of an eHealth platform allows patients and informal caregivers to access information and support from the confines of their own home. During these counseling sessions, which last for about 60 minutes, informal caregivers are invited to join since many of them are in need of information and support. During these sessions, the health care provider assesses the information and support needs of patients and informal caregivers. Many informal caregivers struggle with feelings of psychological distress [37,38]. The role of the informal caregiver in these sessions is twofold. During the consultation sessions, the health care provider addresses the psychological stress in patients and informal caregivers. The emphasis is placed on influencing the participant’s attitude by looking at negative situations and beliefs from a different perspective using shifting perspective and belief selection as the main behavioral change strategies. In addition, the informal caregiver is invited to stimulate healthy behavior in the patient (such as stimulating physical activity) by using “shifting away from unpopular behavior” as strategy. Self-regulation skills, such as goal setting and monitoring, are trained by using cognitive behavioral techniques and motivation interviewing.

#### Health Video Clips

The research group proposed the use of health video clips in addition to behavioral counseling to increase knowledge. These video clips provide basic knowledge about a variety of topics collected in step 1 and 2. Together with a cardiologist (RK), we created video clips about CAD and PCI, CAD and CABG, and medication and side effects. The physical therapist (Ilonka Pol) created a video clip about physical activity, and the psychologist (VJ) created a video clip about psychological distress. These video clips are used as a prerequisite to influence self-regulatory skills. All 5 health video clips are accessible for all patients at any time. The health care provider encourages the patient to access these clips before the coaching sessions. The knowledge obtained in the video clips is discussed during the remote coaching sessions and tailored to the specific situation and needs of the patient or caregiver. The theory-based strategies applied in the health video clips are persuasive communication, imagery, and elaboration. Based on the knowledge obtained by patients in these clips, the health care provider can apply the following strategies during consultation: setting goals, reattribution training, self-monitoring behavior, improving physical and emotional states, and setting graded tasks. A comprehensive overview of all strategies is presented in Multimedia Appendix 5.

### Intervention Plan

#### Remote Coaching Program

The eHealth platform can be accessed by patients using a personal computer or mobile device (smartphone or iPhone). Important prerequisites to using this eHealth platform are ability to use the camera on their device and some basic knowledge about accessing an internet platform. The research group proposes a 3-step coaching trajectory. In the first phase, the patients’ and informal caregivers’ needs are assessed, and additional information and support are provided depending on the patients’ and informal caregivers’ needs. After the first session, the patient can access the health information clips on the eHealth platform to obtain knowledge about a variety of topics (CAD and PCI/CABG, medication and side effects, physical activity, psychological distress, and distressing body signals). During the second session, the health care provider reflects on the obtained knowledge and tailors it to the needs of the patient and informal caregiver if needed. In addition, the health care provider challenges and helps the patient and informal caregiver to formulate short-term goals for the first phase after hospital discharge. In the last session, the health care provider and patient reflect on the patient’s progress since hospital discharge and whether short-term goals are reached. During this session, the health care provider helps the patient in formulating long-term goals for after the CR program. If the patient needs more guidance after the third coaching session, more sessions will be planned. The final intervention plan is presented in Table 2.

**Table 2:** Intervention plan.

Strategies		Content	Target group
**Before the intervention**			
	Assessing patients’ needs	Mandatory workshop—objective: learning to perform a comprehensive assessment to assess the needs of patients and informal caregivers	Health care provider
	Changing the behavior of patients	Mandatory workshop—objective: learning to coach patients and informal caregivers by using evidence-based behavior change techniques (such as motivational interviewing)	Health care provider
**During the intervention**			
	Consulting health care provider	Coaching session 1—objectives: assessing information and support needs of patients and informal caregiver. Getting acquainted with coach, eHealth portal, and CR^a^	Patient and informal caregiver
	Accessing digital health information	Health video clips—objectives: gaining detailed information about CAD,^b^ medication, physical activity, psychological distress, and body signals	Patient and informal caregiver
	Consulting health care provider	Coaching session 2—objectives: health care provider, patient, and informal caregiver reflect on health video clips and formulate short-term goals for the period between hospital discharge and starting CR	Patient and informal caregiver
	Consulting health care provider	Coaching session 3—objectives: health care provider, patient, and informal caregiver reflect on short-term goals and progress. Health care provider and patient formulate long-term goals for during and after CR	Patient and informal caregiver

^a^CR: cardiac rehabilitation.

^b^CAD: coronary artery disease.

**Figure 5:**
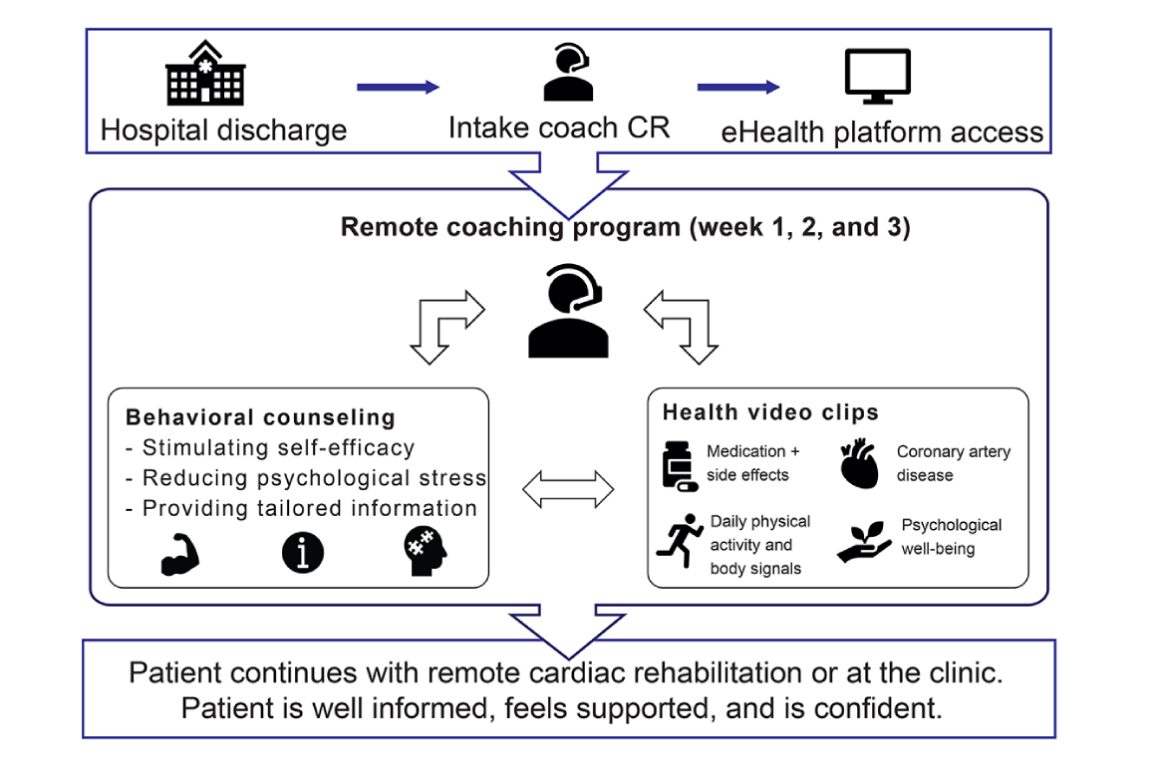
Remote coaching program. CR: cardiac rehabilitation.

In summary, after hospital discharge, patients are approached by a health care provider and gain access to an eHealth platform. During the first session, the patients’ information and support needs are assessed. On the eHealth platform, patients are coached by a health care provider and can access information videos. After the first 4 to 6 weeks, patients continue CR at the CR center or remotely. A flowchart of the intervention is presented in Figure 5.

#### Design and Implementation of the Intervention

After using the IM protocol to create the content of the program, the final intervention was developed. For this intervention, an existing eHealth app from Cardiovitaal Cardiac Rehabilitation Amsterdam was used. After hospital discharge, patients could access this platform to find information and consult with a health care provider before the start of the outpatient CR program. A screenshot of the eHealth platform is presented in Multimedia Appendix 6.

Five health video clips were created together with health care providers who work at the CR center:

- CAD and CABG
- CAD and PCI
- Physical activity after cardiac hospitalization
- Psychological distress after CR
- Medication and side effects

A screenshot of the video clip CAD and PCI is provided in Multimedia Appendix 7, and a screenshot of the video clip physical activity after cardiac hospitalization is provided in Multimedia Appendix 8.

## Discussion

### Principal Findings

The results of this study suggest that patients’ needs after hospital discharge comprise information and support about the following topics: CAD, medication and side effects, daily physical activities, psychological distress, and body signals. In addition, we present a systematic approach to develop an early remote coaching program using the IM protocol.

The overall objective of this remote coaching program is to bridge the gap from hospital discharge to the start of center-based CR by stimulating self-management behavior and influencing the following determinants: knowledge, skills, social support, attitudes, and self-efficacy. We selected theory-based techniques that match these determinants, as research indicates the value of theory-based interventions [57,58]. Moreover, patients were actively involved in the development of this coaching program. To assure adoption of the intervention, patients have been asked to participate in the future refinement of the final intervention in step 4 of the IM approach.

### Comparison With Prior Work

A recent systematic review reports that core components of CR, such as nutrition counseling and psychological and weight management are addressed in one-third of digital CR programs; less than one-third of these programs address management of lipids, diabetes, smoking cessation, and blood pressure [59]. Since our CR program aims to bridge the gap between hospital discharge and the start of CR, we chose to assess the needs of patients in the early phase after hospital discharge. Our study shows that in this phase patients value social support, disease specific information, and information about physical activities and psychological distress. Research shows that health care needs change over time. Nevertheless, knowledge about pathology and how to manage psychological distress remain important even after a 2-year follow-up period [60].

Based on our results, we developed a comprehensive coaching program using remote counseling as the main strategy and considered its positive impact on psychosocial health, physical health, and clinical status [9-11]. In this study, we chose to complement behavioral counseling sessions with educational video clips. The use of video modeling has potential benefits such as facilitation of knowledge acquisition, improving self-care behaviors, and reducing psychological distress [61]. Moreover, video modeling is effective in patients with low health literacy and removes inconsistencies between health care providers [62,63].

Informal caregivers were invited to actively participate in this intervention since research shows that many informal caregivers suffer from psychological distress after their partner’s hospitalization [37-42]. In addition, informal caregivers play an important role in the recovery of the patient; however, hypervigilance in informal caregivers can undermine patients’ health and recovery [64]. It is therefore important to inform and support informal caregivers in their new role. A recent study shows that patients and informal caregivers prefer the same content and delivery formats for digital interventions (eg, health video clips, contact with health care provider) [65]. It is thus expected that informal caregivers can benefit from this remote coaching program.

For this coaching program to be successful, health care providers need to encourage patients to reflect on the obtained knowledge and skills and offer strategies to adopt behavior changes in daily life. Therefore, health care providers should be well trained in applying behavior change techniques and have the ability to build trust in patients.

### Strengths and Limitations

First, we consider the use of the IM protocol a strength since digital health behavior interventions often lack theoretical grounding, as expertise from different scientific areas is often lacking in the design phase [66]. In line with the IM protocol, the development of this intervention was supported by researchers with expertise in various fields (cardiology, physical therapy, psychology, and nursing science), ensuring a firm theoretical approach.

Second, patients’ needs and expectations were taken into account in the early phase of the design process, which contributed to the usability and utility of this intervention [67]. Results from our literature study and interviews indicated that the interval between hospital discharge and CR was too long and that patients wanted to be in contact with a health care provider to receive support and information. It remains unclear, however, if this remote coaching program is applicable for older adult cardiac patients with comorbidities, as they are often reluctant to use eHealth apps [68]. Nevertheless, a recent systematic review shows that older adults (aged 65 years and older) exhibited greater engagement with digital health interventions than younger adults (aged younger than 65 years). Despite the technological barriers, older adults might view digital coaching as social interaction, which is often desired by older adults. In addition, older adults might have more time to engage in digital technologies [69]. In this study, 17 patients with various ages, cardiac diagnoses, and comorbidities were included, and the results of the interviews supported the findings from the literature. It is therefore expected that this intervention is suitable for a wide variety of patients referred for CR. To assure adoption of this intervention by older adults, a thorough evaluation of the feasibility of this intervention should be conducted in step 4 of the IM approach.

Third, the use of a remote intervention is considered a strength since it can resolve several barriers at the patient level (distance to center, transportation) and health care system level (referral problems, limited facilities) [70]. In addition, this study shows that patients are in need of information and support directly after hospital discharge, despite current guidelines, which recommend initiation of CR within 4 to 6 weeks after hospital discharge [4,5]. Delayed participation in CR negatively impacts physical and psychological outcomes, while early initiation of CR positively impacts health outcomes [71-73]. An early remote coaching program starting directly after hospital discharge might help to overcome logistical issues and delays in CR initiation and is therefore well suited for the period between hospital discharge and the start of CR.

Finally, patients in this study were involved in the first steps of the intervention development. Due to the nature of this study, we used a small sample size to explore patients’ needs. However, information obtained from the interviews was supplemented with data from our scoping literature study. We therefore expect that our results are generalizable to the greater cardiac population. Future studies should focus on program refinement (IM step 4) of this remote coaching program and assess its feasibility and effectiveness in studies with larger sample sizes. It would be interesting to assess the effectiveness of this early remote coaching program on symptoms of psychological distress and participation in outpatient CR.

### Conclusion

This study shows that patients with CAD are in need of tailored information and support after hospital discharge. The main areas of information and support are CAD, medication and side effects, physical activity, psychological distress, and body signals. In addition, this study presents the development of a remote intervention, using the IM protocol, to bridge the gap from hospital discharge to the start of CR.

## Appendix

Multimedia Appendix 1 Interview guide.

Multimedia Appendix 2 Extraction table.

Multimedia Appendix 3 Participant quotations.

Multimedia Appendix 4 Determinant matrix.

Multimedia Appendix 5 Program strategy.

Multimedia Appendix 6 Screenshot of eHealth platform.

Multimedia Appendix 7 Screenshot of coronary artery disease and percutaneous coronary intervention.

Multimedia Appendix 8 Screenshot of physical activity after cardiac hospitalization intervention.

## Abbreviations

ACS: acute coronary syndrome
CABG: coronary artery bypass grafting
CAD: coronary artery disease
CR: cardiac rehabilitation
IM: intervention mapping
PCI: percutaneous coronary intervention
PRECEDE: Predisposing, Reinforcing, and Enabling Constructs in Educational Diagnosis and Evaluation
RAAK-PRO: Taskforce for Applied Research
THBC: theory of health behavior change
TPB: theory of planned behavior

## References

[1] Anderson L, Oldridge N, Thompson D, Zwisler A, Rees K, Martin N, Taylor R (2016). Exercise-based cardiac rehabilitation for coronary heart disease: cochrane systematic review and meta-analysis. J Am Coll Cardiol Jan 5.

[2] Lavie CJ, Milani RV, O'Keefe JH, Lavie TJ (2011). Impact of exercise training on psychological risk factors. Prog Cardiovasc Dis.

[3] (2011). Netherlands Society for Cardiology (NVVC) and Netherlands Heart Foundation (NHS) (both Guidelines on Cardiac Rehabilitation 2004) and Working Group PAAHR (partial revision 2011).

[4] Fell J, Dale V, Doherty P (2016). Does the timing of cardiac rehabilitation impact fitness outcomes? An observational analysis. Open Heart.

[5] Sunamura M, Ter Hoeve N, Geleijnse ML, Steenaard RV, van den Berg-Emons HJG, Boersma H, van Domburg RT (2017). Cardiac rehabilitation in patients who underwent primary percutaneous coronary intervention for acute myocardial infarction: determinants of programme participation and completion. Neth Heart J.

[6] Rao A, Zecchin R, Newton P, Phillips J, DiGiacomo M, Denniss A, Hickman L (2020). The prevalence and impact of depression and anxiety in cardiac rehabilitation: a longitudinal cohort study. Eur J Prev Cardiol.

[7] Ghisi GLDM, Abdallah F, Grace SL, Thomas S, Oh P (2014). A systematic review of patient education in cardiac patients: do they increase knowledge and promote health behavior change?. Patient Educ Couns.

[8] Berra K (2011). Does nurse case management improve implementation of guidelines for cardiovascular disease risk reduction?. J Cardiovasc Nurs.

[9] Veen EV, Bovendeert JFM, Backx FJG, Huisstede BMA (2017). E-coaching: new future for cardiac rehabilitation? A systematic review. Patient Educ Couns.

[10] Rawstorn JC, Gant N, Meads A, Warren I, Maddison R (2016). Remotely delivered exercise-based cardiac rehabilitation: design and content development of a novel mHealth platform. JMIR Mhealth Uhealth.

[11] Keesman M, Janssen V, Kemps H, Hollander M, Reimer WSO, Gemert-Pijnen LV, Hoes A, Kraaij W, Chavannes N, Atsma D, Kraaijenhagen R, Evers A, BENEFIT consortium (2019). BENEFIT for all: an ecosystem to facilitate sustained healthy living and reduce the burden of cardiovascular disease. Eur J Prev Cardiol.

[12] Janssen V, Kraaijenhagen R (2016). Do-it-yourself health care: a three-step approach to supporting patient self-management in clinical practice.

[13] Majid U, Kim C, Cako A, Gagliardi AR (2018). Engaging stakeholders in the co-development of programs or interventions using Intervention Mapping: a scoping review. PLoS One.

[14] Bartholomew LK, Parcel GS, Kok G (1998). Intervention mapping: a process for developing theory- and evidence-based health education programs. Health Educ Behav.

[15] Binkley C, Johnson K (2013). Application of the PRECEDE-PROCEED planning model in designing an oral health strategy. J Theory Pract Dent Public Health.

[16] Frederiksen L, Phelps S Literature review for education and nursing graduate students.

[17] Keessen P, Latour CHM, van Duijvenbode ICD, Visser B, Proosdij A, Reen D, Scholte Op Reimer WJM (2020). Factors related to fear of movement after acute cardiac hospitalization. BMC Cardiovasc Disord.

[18] Forster A, Brown L, Smith J, House A, Knapp P, Wright JJ, Young J (2012). Information provision for stroke patients and their caregivers. Cochrane Database Syst Rev.

[19] Wachters-Kaufmann C, Schuling J, The H, Meyboom-de Jong B (2005). Actual and desired information provision after a stroke. Patient Educ Couns.

[20] Alkubati SA, Al-Zaru IM, Khater W, Ammouri AA (2013). Perceived learning needs of Yemeni patients after coronary artery bypass graft surgery. J Clin Nurs.

[21] Eshah N (2011). Jordanian acute coronary syndrome patients' learning needs: implications for cardiac rehabilitation and secondary prevention programs. Nurs Health Sci.

[22] Almaskari AA, Al Noumani H, Al-Omari K, Al Maskari MA (2019). Patients' and nurses' perceptions of post-coronary artery bypass graft learning needs in two Omani hospitals. Sultan Qaboos Univ Med J.

[23] Omari F, Al-Zaru I, AL-Yousef RH (2013). Perceived learning needs of Syrian patients postcoronary artery bypass graft surgery. J Clin Nurs.

[24] Akbari M, Celik S (2015). The effects of discharge training and counseling on post-discharge problems in patients undergoing coronary artery bypass graft surgery. Iran J Nurs Midwifery Res.

[25] Mosleh SM, Eshah NF, Almalik MM (2017). Perceived learning needs according to patients who have undergone major coronary interventions and their nurses. J Clin Nurs.

[26] Krannich J, Herzog M, Weyers P, Lueger S, Faller H, Bohrer T, Lange V, Elert O, Leyh R (2009). Patients' needs during hospitalization in a cardiac surgery unit before and after coronary artery bypass graft surgery. Thorac Cardiovasc Surg.

[27] Lie I, Bunch EH, Smeby NA, Arnesen H, Hamilton G (2012). Patients' experiences with symptoms and needs in the early rehabilitation phase after coronary artery bypass grafting. Eur J Cardiovasc Nurs.

[28] Kilonzo B, O'Connell R (2011). Secondary prevention and learning needs post percutaneous coronary intervention (PCI): perspectives of both patients and nurses. J Clin Nurs.

[29] Jickling JL, Graydon JE (1997). The information needs at time of hospital discharge of male and female patients who have undergone coronary artery bypass grafting: a pilot study. Heart Lung.

[30] Kattainen E, Meriläinen P, Jokela V (2004). CABG and PTCA patients' expectations of informational support in health-related quality of life themes and adequacy of information in 1-year follow-up. Eur J Cardiovasc Nurs.

[31] Polikandrioti M, Goudevenos J, Michalis LK, Ioannis K, Elpida G, Kostas K, Elisaf M (2015). Correlation between the type of acute coronary syndrome with the needs of hospitalized patients. Glob J Health Sci.

[32] Czar ML, Engler MM (1997). Perceived learning needs of patients with coronary artery disease using a questionnaire assessment tool. Heart Lung.

[33] Kähkönen O, Kankkunen P, Miettinen H, Lamidi M, Saaranen T (2017). Perceived social support following percutaneous coronary intervention is a crucial factor in patients with coronary heart disease. J Clin Nurs.

[34] Aazami S, Jaafarpour M, Mozafari M (2016). Exploring expectations and needs of patients undergoing angioplasty. J Vasc Nurs.

[35] Pier C, Shandley KA, Fisher JL, Burstein F, Nelson MR, Piterman L (2008). Identifying the health and mental health information needs of people with coronary heart disease, with and without depression. Med J Aust.

[36] Gao F, Yao K, Tsai C, Wang K (2009). Predictors of health care needs in discharged patients who have undergone coronary artery bypass graft surgery. Heart Lung.

[37] Antonakoudis H, Kifnidis K, Andreadis A, Fluda E, Konti Z, Papagianis N, Stamou H, Anastasopoulou E, Antonakoudis G, Poulimenos L (2006). Cardiac rehabilitation effects on quality of life in patients after acute myocardial infarction. Hippokratia.

[38] Halm MA (2017). Age and gender influences on the needs, concerns and strategies of CABG caregivers. Heart Lung.

[39] Lukkarinen H, Kyngäs H (2016). Experiences of the onset of coronary artery disease in a spouse. Eur J Cardiovasc Nurs.

[40] Moore SM (1994). Psychologic distress of patients and their spouses after coronary artery bypass surgery. AACN Clin Issues Crit Care Nurs.

[41] Rolley J, Smith J, DiGiacomo M, Salamonson Y, Davidson P (2011). The caregiving role following percutaneous coronary intervention. J Clin Nurs.

[42] Halm MA (2016). Specific needs, concerns, strategies and advice of caregivers after coronary artery bypass surgery. Heart Lung.

[43] Askham J, Kuhn L, Frederiksen K, Davidson P, Edward K, Worrall-Carter L (2010). The information and support needs of Faroese women hospitalised with an acute coronary syndrome. J Clin Nurs.

[44] Perk J, Hambraeus K, Burell G, Carlsson R, Johansson P, Lisspers J (2015). Study of Patient Information after percutaneous Coronary Intervention (SPICI): should prevention programmes become more effective?. EuroIntervention.

[45] Gentz CA (2000). Perceived learning needs of the patient undergoing coronary angioplasty: an integrative review of the literature. Heart Lung.

[46] Bonnet F, Irving K, Terra J, Nony P, Berthezène F, Moulin P (2005). Anxiety and depression are associated with unhealthy lifestyle in patients at risk of cardiovascular disease. Atherosclerosis.

[47] Mühlbacher AC, Bethge S, Kaczynski A (2016). Treatment after acute coronary syndrome: analysis of patient's priorities with analytic hierarchy process. Int J Technol Assess Health Care.

[48] Valaker I, Norekvål T, Råholm M, Nordrehaug JE, Rotevatn S, Fridlund B, CONCARD Investigators (2017). Continuity of care after percutaneous coronary intervention: the patient's perspective across secondary and primary care settings. Eur J Cardiovasc Nurs.

[49] de Melo Ghisi GL, Grace SL, Thomas S, Evans MF, Sawula H, Oh P (2014). Healthcare providers' awareness of the information needs of their cardiac rehabilitation patients throughout the program continuum. Patient Educ Couns.

[50] Pedersen M, Overgaard D, Andersen I, Baastrup M, Egerod I (2017). Experience of exclusion: a framework analysis of socioeconomic factors affecting cardiac rehabilitation participation among patients with acute coronary syndrome. Eur J Cardiovasc Nurs.

[51] Anderson DR, Emery CF (2014). Irrational health beliefs predict adherence to cardiac rehabilitation: a pilot study. Health Psychol.

[52] Svavarsdóttir M, Sigurðardóttir Á, Steinsbekk A (2016). Knowledge and skills needed for patient education for individuals with coronary heart disease: the perspective of health professionals. Eur J Cardiovasc Nurs.

[53] Svavarsdóttir MH, Sigurdardottir AK, Steinsbekk A (2016). What is a good educator? A qualitative study on the perspective of individuals with coronary heart disease. Eur J Cardiovasc Nurs.

[54] Bäck M, Öberg B, Krevers B (2017). Important aspects in relation to patients' attendance at exercise-based cardiac rehabilitation—facilitators, barriers and physiotherapist's role: a qualitative study. BMC Cardiovasc Disord.

[55] Laranjo L, Syed-Abdul S, Gabarron E, Lau A (2016). Social media and health behavior change. Participatory Health Through Social Media.

[56] Ajzen I (2005). Attitudes, Personality and Behavior, 2nd edition.

[57] Kok G, Gottlieb NH, Peters GY, Mullen PD, Parcel GS, Ruiter RAC, Fernández ME, Markham C, Bartholomew LK (2016). A taxonomy of behaviour change methods: an Intervention Mapping approach. Health Psychol Rev.

[58] Joseph RP, Daniel CL, Thind H, Benitez TJ, Pekmezi D (2016). Applying psychological theories to promote long-term maintenance of health behaviors. Am J Lifestyle Med.

[59] Wongvibulsin S, Habeos EE, Huynh PP, Xun H, Shan R, Porosnicu Rodriguez KA, Wang J, Gandapur YK, Osuji N, Shah LM, Spaulding EM, Hung G, Knowles K, Yang WE, Marvel FA, Levin E, Maron DJ, Gordon NF, Martin SS (2021). Digital health interventions for cardiac rehabilitation: systematic literature review. J Med Internet Res.

[60] Greco A, Cappelletti ER, Monzani D, Pancani L, D'Addario M, Magrin ME, Miglioretti M, Sarini M, Scrignaro M, Vecchio L, Fattirolli F, Steca P (2016). A longitudinal study on the information needs and preferences of patients after an acute coronary syndrome. BMC Fam Pract.

[61] Krouse HJ (2001). Video modelling to educate patients. J Adv Nurs.

[62] Sobel RM, Paasche-Orlow MK, Waite KR, Rittner SS, Wilson EAH, Wolf MS (2009). Asthma 1-2-3: a low literacy multimedia tool to educate African American adults about asthma. J Community Health.

[63] Gagliano ME (1988). A literature review on the efficacy of video in patient education. J Med Educ.

[64] Campbell L, La Guardia J, Olson J, Zanna M (2012). The Science of the Couple, 1st ed.

[65] Sankaran S, Bonneux C, Dendale P (2018). Bridging patients’ needs and caregivers’ perspectives to tailor information provisioning during cardiac rehabilitation. Proc 32nd Int BCS Hum Comput Interact Conf.

[66] Taj F, Klein M, van Halteren A (2019). Digital health behavior change technology: bibliometric and scoping review of two decades of research. JMIR Mhealth Uhealth.

[67] Demiris G, Afrin LB, Speedie S, Courtney KL, Sondhi M, Vimarlund V, Lovis C, Goossen W, Lynch C (2008). Patient-centered applications: use of information technology to promote disease management and wellness. A white paper by the AMIA knowledge in motion working group. J Am Med Inform Assoc.

[68] Graham SA, Stein N, Shemaj F, Branch OH, Paruthi J, Kanick SC (2021). Older adults engage with personalized digital coaching programs at rates that exceed those of younger adults. Front Digit Health.

[69] Nymberg VM, Bolmsjö B, Wolff M, Calling S, Gerward S, Sandberg M (2019). "Having to learn this so late in our lives…" Swedish elderly patients' beliefs, experiences, attitudes and expectations of e-health in primary health care. Scand J Prim Health Care.

[70] Brouwers RWM, van Exel HJ, van Hal JMC, Jorstad HT, de Kluiver EP, Kraaijenhagen RA, Kuijpers PMJC, van der Linde MR, Spee RF, Sunamura M, Uszko-Lencer NHMK, Vromen T, Wittekoek ME, Kemps HMC, Committee for Cardiovascular Prevention and Cardiac Rehabilitation of the Netherlands Society of Cardiology (2020). Cardiac telerehabilitation as an alternative to centre-based cardiac rehabilitation. Neth Heart J.

[71] Pizzorno M, Desilvestri M, Lippi L, Marchioni M, Audo A, de Sire A, Invernizzi M, Perrero L (2021). Early cardiac rehabilitation: could it improve functional outcomes and reduce length of stay and sanitary costs in patients aged 75 years or older? A retrospective case-control study. Aging Clin Exp Res.

[72] Zhang Y, Lu Y, Tang Y, Yang D, Wu H, Bian Z, Xu J, Gu C, Wang L, Chen X (2016). The effects of different initiation time of exercise training on left ventricular remodeling and cardiopulmonary rehabilitation in patients with left ventricular dysfunction after myocardial infarction. Disabil Rehabil.

[73] Sumner J, Böhnke J, Doherty P (2018). Does service timing matter for psychological outcomes in cardiac rehabilitation? Insights from the National Audit of Cardiac Rehabilitation. Eur J Prev Cardiol.

