# Supplementary material for: Design of a Remote Coaching Program to Bridge the Gap From Hospital Discharge to Cardiac Rehabilitation: Intervention Mapping Study
Source: JMIR Cardio. 2022 May 25;6(1):e34974. doi: 10.2196/34974 (PMC9178457; doi:10.2196/34974)
Supplement: Multimedia Appendix 1 [file cardio_v6i1e34974_app1.docx]

Multimedia Appendix 1. Interview guide.

| **Themes** | **Topics** |
| --- | --- |
| *Medication* | Type of medication, Effect of medication, Dose of medication, Interactions, Side effects, other |
| *Surroundings* | Support, Partner, Sexuality, Support, Other |
| *Disease knowledge* | Cardiac illness, type of intervention, prognosis |
| *Daily activities* | Return to work, Leisure |
| *Lifestyle* | Smoking, Diet, Alcohol, Physical activity |
| *Disease impact* | Dealing with stress and worry, Anxiety, Depression, Body signals, Sleep |
| *Future* | Course of disease, Chance of recurring cardiac event, Where to find information and who to call |
