# Supplementary material for: Design of a Remote Coaching Program to Bridge the Gap From Hospital Discharge to Cardiac Rehabilitation: Intervention Mapping Study
Source: JMIR Cardio. 2022 May 25;6(1):e34974. doi: 10.2196/34974 (PMC9178457; doi:10.2196/34974)
Supplement: Multimedia Appendix 2 [file cardio_v6i1e34974_app2.docx]

Multimedia Appendix 2. Extraction table.

| **Reference** | **Country** | **Study design** | **Participants / studies** | **Data collection** | **Main finding(s)** |
| --- | --- | --- | --- | --- | --- |
| Aazami et al. (2016) | Iran | Cross sectional qualitative study | 18 PCI patients | Semi structured interviews after hospital discharge | This study showed that there is uncertainty and insecurity in patients who undergo angioplasty. This study emphasizes the need for procedural knowledge, social support. |
| Akbari et al. (2015) | Iran | Semi-experimental study | 100 CABG patients | Intervention: discharge training 6 weeks post discharge with booklet before surgery and 6 weeks after.  Control group: usual care: prescribing medication, wound care and checking vital signs. | This study showed that patients in the intervention group experienced fewer problems concerning:  Job status (P<0.03)  Chronic illness (P<0.03)  Respiratory difficulties (P<0.05)  Blood pressure (P<0.03)  Palpitation (P<0.001)  Wound healing (P<0.001) |
| Alkubati et al (2021) | Jordan | Cross sectional | 120 CABG patients | Cardiac Patients Learning Needs Inventory (CPLNI) before hospital discharge | This study showed that CABG patients have high information needs within 24-48 hours before hospital discharge.  Patients wanted information about: (1) chest and leg wound care  (2) medication information,  (3) complications.  Male patients needed more information than female patients (p=0.004). Younger (p=0.021) and middle-aged (p=0.032) patients needed more information than older ones. Highly educated (p=0.000) and school-level (p=0.002) patients needed more information than those in the uneducated group. Working patients needed more information than non-working ones (p=0.000). |
| Almaskari et al. (2019) | Oman | Cross sectional | 90 CABG patients and 90 nurses | Modified Cardiac Patients Learning Need Inventory (MCPLNI) | This study showed that nurses perceived information about chest and leg wound care, medications and complications as patients' most important learning need. Patients perceived a need for post-CABG learning related to chest and leg wound care, complications and medication as most important, while learning related to physical activity received a moderate priority. |
| Anderson et al. (2014) | United States | Cross sectional | 61 patients referred for CR | Participants completed a baseline questionnaire including measures of depression and irrational health beliefs | This study showed that older age (p < .05) and higher income (p < .05) were associated with better CR adherence, but CR adherence was lower among African Americans than Caucasians (p < .01). Depression was not related to adherence (p = .78), but irrational health beliefs predicted CR adherence (β=-0.29, P <0.5) |
| Antonakoudis (2006) | Greece | Non randomized trial | 110 ACS  Group A participated in cardiac rehabilitation  Group B did not participate in cardiac rehabilitation | Velasco-Del Barrio questionnaire for Health Related Quality of Life | This study showed that a significant difference was found between Group A and B regarding the parameters symptoms (17±6.8 vs 22±6.5, p<0.001) and social behavior (21±4.2 vs 23±5.5, p<0.0001). |
| Askham et al. (2010) | Denmark and Faroe Islands | Cross sectional | 8 female ACS patients | Semi-structured interviews 3-4 weeks after hospital discharge. | This study presents the following themes related to needs.  (1) 'Discharged home, now what?': illustrated the difficulties experienced by these women following discharge and  (2) risk factor management and lifestyle adjustments: related to perceptions relating to risk, loss, recovery and support. |
| Bäck et al. (2017)  . | Sweden | Cross sectional | 16 patients with ACS | Interviews, analyzed with inductive content analysis | This study presents aspects that influence patients’ attendance at cardiac rehabilitation.   1. previous experience of exercise, 2. needs in the acute phase, 3. important prerequisites for attending exercise-based CR and (iv) future ambitions. |
| Bonnet et al. (2005) | France | Cross sectional | 1612 patient at risk for cardiovascular disease | Assessment of unhealthy lifestyle factors (physical inactivity, smoking and poor diet) and symptoms of anxiety and depression. | This study showed that both anxiety and depression appeared as independent determinant of an unhealthy lifestyle in both sexes, with a stronger influence for depression. Depression and to a lesser extent anxiety are associated with a cluster of unhealthy behaviors in subjects at risk of cardiovascular disease, suggesting the difficulty of modifying lifestyle in patients with anxious-depressive disorders. |
| Czar et al. (1997) | United States | Longitudinal study | 28 patients treated with PCI after or ACS or Stable Angina pectoris. | Learning needs questionnaire at hospital discharge and first visit at the clinic after discharge | This study showed that the most important learning needs were: symptom recognition, cardiac anatomy and physiology, and medications. |
| de Melo Ghisie al (2014) | Canada | Cross sectional | 306 CR patients and 28 CR providers | Information Needs in Cardiac Rehabilitation (INCR) questionnaire | This study showed that low-income CR participants had significantly greater information needs than high-income participants. CR providers were cognizant of patient information needs, except patients did desire more information on diagnosis and treatment than providers perceived |
| Eshah et al. (2011). | Jordan | Cross sectional | 150 patients with acute coronary syndrome | The Patient Learning Needs Scale (PLNS) was collected at the cardiac care unit | This study showed that highest information needs concerned: medication and treatment and activities of daily living. Older patients, and those with low SES, requested less information than others did. |
| Forster et al. (2021) | Various | Systematic review | 33 studies involving 5255 stroke-survivors and 3134 informal caregivers | 2 reviewers independently assessed trial eligibility and risk of bias. Interventions were categorized: active information provision included: active participation with subsequent opportunities for clarification and reinforcement; passive information provision provided no systematic follow-up or reinforcement procedure. Overall certainty of evidence was assessed with GRADE. | This study showed that: active information vs. passive information provision may improve stroke-survivor knowledge and quality of life (SMD) 0.41, 95% confidence interval (CI) 0.17 to 0.65, and may reduce anxiety ((RR) 0.85, 95% CI 0.68 to 1.06; 5 studies, 1132 participants) and depression.  (RR 0.83, 95% CI 0.68 to 1.01; 6 studies, 1315 participants) |
| Gao et al. (2009) | Taiwan | Cross sectional | 103 CABG patients | Symptom Distress Inventory and Health Care Needs Inventory within 1 month after discharge | This study showed that the most important predictors for overall health care needs were length of hospital stay after surgery, symptom distress, and gender |
| Gentz et al. (2000) | USA | Systematic Review | 19 studies involving patients learning needs and concern after PCI. | Studies were identified in CINAHL and MEDLINE | This study showed that Informational knowledge, such as risk factor education and survival management, were considered of high importance. The majority of subjects modified their behavior, and the most common modification was in diet. Both learned knowledge and lifestyle changes decreased over time. Self-efficacy expectations and levels of anxiety were predictors of behavior changes and knowledge retention in the early recovery period after the coronary angioplasty procedure. |
| Halm (2017) | United states | Cross sectional | 32 CABG caregivers | Interviews in the first 3 months after hospital discharge | This study showed that age-specific themes related to motivating exercise (<70), applying support hose, and self-reliance (>70). Gender-specific themes related to incision worries and transportation (all females); regulating blood sugars, vital sign anxiety, hurrying back, reinforcing healthy behaviors, and being there (females <70); meal planning, coordinating appointments, and anger over self-care reminders (females >70); caregiver relief, inconsistent information, and doing it all (all males); upsetting scars (males <70), and lost sleep (male >70). |
| Halm et al. (2016) | United states | Cross sectional | 32 Caregivers of patients that underwent CABG | Telephone interviews 3 months post discharge | This study showed that the needs of informal caregivers of patients that underwent CABG comprise information and support about:  (1) 'knowing what I'm supposed to be doing'  Information about:  (2) medication  (3)mobility  (4) symptom monitoring  (5)memory  (6) appetite  (7) emotional spirits  (8) finances |
| Jickling et al. (1997)  . | Canada | Cross sectional | 20 men and 20 women with a 1st time Elective CABG | Patient Learning Needs Scale and open-ended question directly after hospital discharge. | This study showed that no differences were found in information needs between men and women. The highest areas of information needs included: treatment and complications, activities, medications, and enhancing quality of life. |
| Kähkönen et al. (2017)  . | Finland | Cross sectional | 416 patient with CAD | Social Support of People with Coronary Heart Disease questionnaire after hospital discharge | This study showed that healthcare professionals should pay extra attention to women, single patients, physically inactive patients, those demonstrating a lower level of education, those with a longer duration of CHD, and respondents without previous acute myocardial infarction. Continuum of care and counselling are important to ensure especially among them. |
| Kattainen et al (2004) | Finland | Longitudinal study | 439 Men and176 women with elective PCI and CABG | The data were collected by structured interview before coronary artery procedures and by mailed questionnaires 6 and 12 months afterwards | This study showed that patients in both groups needed information about recovery and psychosocial functioning before and after the treatments. Women in the PTCA group needed more informational support than men before procedures, while men needed more support afterwards. |
| Keessen et al. (2020) | Netherlands | Cross sectional | 16 patients with acute coronary syndrome, atrial fibrillation or acute heart failure | Semi structured interviews after hospital discharge. | This study showed that after hospital discharge patients are in need of:   1. Tailored information 2. Support health care provider |
| Kilonzo et al. (2011) | Ireland | Cross sesctional | 33 PCI patients 13 nurse | Survey directly after PCI | This study showed that patients preferred information and support about: Disease-specific items, physical action, psychosocial and emotional information. Cardiac nurses perceived physical action as least important and focused more on psychosocial and emotional issues. |
| Krannich et al (2009) | Germany | Longitudinal study with 2 time points | 70 CABG patients | Patients filled in their needs 48 hours after hospital discharge and 10 days after hospital discharge. | This study showed that before CABG, patients rated the need for "preparation for surgery", and after CABG the need for "information about the correct handling of drugs", as the most important. The rating of "information about the correct handling of drugs" showed a significant increase after CABG surgery ( T(69) = - 3.46; P < 0.001) and the need for a "letter with the latest scientific information on heart disease" was significantly reduced during this period ( T(69) = 2.07; P = 0.04) |
| Lie I, et al. (2012) | Norway | Longitudinal study | 93 CABG patients | 2 and 4 weeks after CABG | This study showed that needs of patients were characterized by a substantial amount of uncertainty and worries related to what to expect and what was normal for postoperative pain, assessment and sensation of surgical site, different experiences with physical activity/exercise, uncertainty about medications, difficulties with sleep pattern, irritability, postoperative complications, uncertainty about return to work, and insufficient information at discharge. |
| Lukkarinen et al. (2003) | Finland | Cross sectional | 146 informal caregivers of patient that underwent PCI or CABG | Open ended questions questionnaire | This study showed that informal caregivers: had to monitor for symptoms, treat symptoms, take care, understand and support. They had to assume responsibility for everyday life. They felt themselves to be alone in that situation. They did not receive support from health care providers. All informants felt uncertainty due to financial problems, poorly planned care and unexpected changes in the course of the disease. |
| Moore et al. (1994) | United states | Cross sectional | CABG Patients and spouses | Profiles of mood states questionnaire 6 weeks post CABG | This study showed that spouses experienced more psychological distress than patients at 6 weeks after surgery. Younger spouses reported more anger than older spouses. |
| Mosleh et al (2017) | Jordan | Cross sectional study | 365 cardiac patients (PCI and CABG) and 166 cardiac nurses | Patients completed the Patient Learning Needs Scale (PLNS) within 24-48 hours after hospital discharge. | This study showed that the top-priority learning needs according to both patients and cardiac nurses was information on wound care and medication. In contrast, the lowest-priority learning need was physical activity. Nurses perceived information about physical activity as most needed to patients, whereas patients perceived information about medications, postintervention complications and postintervention concerns as mostly needed. |
| Mühlbacher et al (2016). | Germany | Cross sectional | 623 ACS patients | Analytic Hierarchical Process to evaluate important patient outcomes. | This study showed that patients showed a clear priority for the attribute "reduction of mortality risk" (weight: 0.402). The second most preferred attribute was the "prevention of a new myocardial infarction" (weight: 0.272), followed by "side effect: dyspnea" (weight: 0.165) and "side effect: bleeding" (weight: 0.117). The "frequency of intake" was the least important attribute (weight: 0.044). |
| Omari et al  (2014) | Syria | Cross sectional | 135 CABG patients | Modified Cardiac Patients Learning Needs Inventory (MCPLNI) | This study showed that information about chest and leg wound care, complications, medication and physical activity were the most important learning needs. There were significant differences between patients' perceptions of learning needs and their age, chronic illnesses and their working status. |
| Pedersen et al. (2017) | Denmark | Cross sectional | 24 ACS patients and 12 informal caregivers. | Semi structures interviews with 12 patients that completed the full CR program and 12 that did not complete CR. | This study showed that non-participation in CR was explained by the following themes: exclusion by time and place, exclusion by health beliefs, exclusion from counseling, exclusion by alienation, and exclusion of relatives. |
| Perk et al. (2015) | Sweden | Cross sectional | 1,073 PCI patients | Questionnaire after revascularization about: patient's attribution of the cause of the cardiac event, perception of the information provided by physicians and nurses, and a self-assessment of changes in lifestyle post PCI regarding tobacco, physical activity, food habits and stress. | This study showed that non-modifiable risk factors (age, heredity) were attributed a higher rate as the cause of disease compared to modifiable factors (smoking, physical activity, food habits). Most patients (67%) perceived they were cured, and 38% perceived from the given information that there was no need to change their habits. A mere 27% reported that they still had cardiovascular disease and needed behavioral change. After PCI, 16% continued to use tobacco; half of these were offered smoking cessation support. In spite of an 80% referral rate to cardiac rehabilitation, one out of two patients did not enroll. Fewer than half were regularly physically active. |
| Pier et al. (2008) | Australia | Cross sectional study | 14 patients with CAD | Semi-structured interviews | This study showed that the most prominent information needs included identification and management of risk-related physical symptoms, and psychosocial information, most notably to enhance patients' social support. Patients considered this information important for alleviating health anxiety and negative affect. |
| Polikandrioti et al. (2015) | Greece | Cross sectional study | 454 hospitalized patients with ACS | Needs of hospitalized patients with coronary artery disease questionnaire at hospital discharge. | This study showed that the type of ACS was statistically significant correlated with the place of residence (p=0.002), management of disease (p<0.001) and prior experience of hospitalization (p=0.003). All six needs were statistically significantly correlated with the type of ACS, (p<0.001 for the need for support and guidance, p<0.001 for the need to be informed from the medical and nursing staff, p<0.001 for the need for being in contact with other patient groups, and ensuring communication with relatives, p<0.001 for the need for individualized treatment and for the patient's personal participation to his/her treatment, p<0.001 for the need to meet the emotional needs and physical needs and p=0.010 for the need to trust the medical and nursing staff). |
| Rolley et al. (2011) | Australia | Cross sectional | 18 Caregivers of patients that underwent PCI. | Focus group | This study showed that the needs of caregivers comprised:  (1) a gendered approach to health, illness and caring;  (2) shock, disbelief and the process of adjustment following PCI;  (3) challenges and changes of the carer-patient relationship and  (4) the needs of the carer for support and information |
| [Svavarsdóttir](https://pubmed.ncbi.nlm.nih.gov/?term=Svavarsd%C3%B3ttir+MH&cauthor_id=26588939) et al. (2015) | Iceland and Norway | Cross sectional | 17 PCI patients | Interviews with systematic text condensation. | This study showed that patients saw a good educator as one who they feel is trustworthy and who individualizes the education to patients' needs and context and translates general information to their personal situation in lay language. Building trust was dependent on the patients' perceiving the educator to be knowledgeable and good at connecting with the individual patient, so that the patients feel they are being treated as a whole person with equality and respect. |
| [Svavarsdóttir](https://pubmed.ncbi.nlm.nih.gov/?term=Svavarsd%C3%B3ttir+MH&cauthor_id=26588939) et al. (2016) | Iceland and Norway | Cross sectional | 19 health care providers (nurses, physiotherapists and cardiologists) | Interviews, analyzed with systematic text condensation. | This study showed:  Knowledge and skills needed for being a good educator defined by health care providers:  This includes being able to establish interpersonal relationships with patients, capturing their learning needs, facilitating an effective dialogue and providing individualized patient centered education and lifestyle counselling. |
| Valaker et al (2017)  . | Norway | Cross sectional | 22 PCI patients | In depth interviews with patients 6-8 weeks after PCI to explore continuity of care | This study showed that patients were not receiving adequate instruction and information on how to integrate health information. Patients also needed help to facilitate connections to community-based resources and to schedule clear follow-up appointments. |
| Wachters-Kaufmann et al. (2005) | Netherlands | Longitudinal study | 33 stroke survivors and 27 caregivers | Telephone survey at 3 and 12 months after hospital discharge | This study showed that patients and caregivers prefer to receive information within 24h and to be informed about, and be given, relevant written information. The information given by the various professional stroke care-providers could be better coordinated. The role of the GP as an information provider lagged quite a long way behind. |
