# Supplementary material for: Design of a Remote Coaching Program to Bridge the Gap From Hospital Discharge to Cardiac Rehabilitation: Intervention Mapping Study
Source: JMIR Cardio. 2022 May 25;6(1):e34974. doi: 10.2196/34974 (PMC9178457; doi:10.2196/34974)
Supplement: Multimedia Appendix 3 [file cardio_v6i1e34974_app3.docx]

Multimedia Appendix 3. Quotations.

| **Categories** | **Subcategories** | **Quotes** |
| --- | --- | --- |
| **Distressing body signals** | Fatigue | *`I get tired so quickly’ (P15)* |
|  | Wound pain / chest pain | *`I don’t want to feel that pain anymore’ (P11)* |
|  | Hypervigilance | *`I don’t dare to do anything’ (P16)* |
|  |  | *`If my heart skips a beat, I don’t do anything, I’m really anxious to become short of breath’ (P10)* |
|  | Fear of bodily sensations | *`I monitor my heart rate’ (P17)* |
|  |  | *`And when I am sitting on the couch, or walking around, I feel every sting. That frightens me’ (P8)* |
| **Psychological problems** | Anxiety | *`I have many panic attacks’ (P12)* |
|  | Depression | *`My whole outlook on life has changed after my event’. (P12)* |
|  |  | *`Nothing will be the same again’ (P16)* |
| **Lack of information hospital discharge** | Medication | *`At hospital discharge the physician gave me a whole list with medication and then said I could go home, that’s all he said, this made me insecure.’ (P10)* |
|  | Side effects medication | *`I felt a weird pressure on my chest, like my heart skipped a beat. I panicked, so I went back to the emergency room where they examined me. Afterwards they told me it was a side effect of metropolol’ (P10)* |
|  |  | *`I would like to know why I have to take those pills, I had lots of side effects’ (P9)* |
|  |  | *`I missed information about medication and side effects’. At first, I felt better than after my myocardial infarction, until the statins began to work. I sat on the couch like a dead bird, muscle ache everywhere and unable to move’ (P8)* |
|  | Physical activity | *`I don’t know if I can do any physical activity and if I injure my body if do physical activity’ (P4)* |
|  |  | *`They did not gave me any information about what I could and could not do, looking back I find this very bad’(P4)* |
|  |  | *`What is my heart able to handle? Not knowing this, is very annoying’ (P2)* |
|  |  | *`I just want some simple information about what I can and cannot do, can I walk the stairs? Can I drive my car?’(P11)* |
|  |  | *‘I want be confident again that I don’t injure myself, by walking stairs or walking for miles’ (P2)* |
|  | Inconsistent information | *`One health care provider tells me this the other tells me that’ (P1)* |
|  |  | *`What does it mean to take it easy?’ (P2)* |
|  |  | *`It’s not a small thing, having a heart attack. In the hospital you don’t know what’s going on and when you leave you still don’t know’ (P8)* |
|  | Cardiac event | *`They didn’t tell me anything at the hospital and it passed by so quick. Who says I won’t suffer another myocardial infarction’ (P10)* |
|  |  | *`I really missed talking to my physician about what had happened to my heart before I left the hospital’ (P7)* |
|  | Procedure | *`Someone told me that a stent can shift within the artery, if I’m not feeling well, I think about this’ (P8)* |
| **Passive coping style** | Inactivity | *`I rather be in bed all the time’ (P16)* |
|  |  | *`I did not do anything for six weeks, I’m just staying in bed and on the couch, I can’t do much more’ (P9)* |
| **Healthcare system** | Unclear communication Health care provider | *`I did not know I would get a pacemaker’ (P14)* |
|  |  | *`One health care provider told me I could cycle again but then the other told me not to’ (P14)* |
|  | Time until cardiac rehabilitation | *`I think the time between discharge and CR is too long’. (P12)* |
|  |  | *`Four weeks is quite long while waiting for cardiac rehabilitation’ (P10)* |
|  | Relevance of cardiac rehabilitation | *`What is there to rehabilitate about the heart?’ (P2)* |
|  | Negative experience Hospital | *`I didn’t feel well for a long time but they did not listen to me’. (P15)* |
|  |  | *`Everyone was so busy, they did not have time for everyone’ (P15)* |
|  |  | *`I was left alone in a bed and did not see anyone during my stay at the nursing ward. There was no one that came to me to ask me how I was doing and if I was afraid. Just some human contact would make it so much better’ (P8)* |
|  |  | *`The referral to cardiac rehabilitation went completely wrong. It took ages before it was clear where I needed to go and what was expected. Thinking about this makes me short of breath again’ (P4)* |
|  | Trusting caregivers | *`Trusting caregivers, cardiologist, nurse practitioners, physiotherapists is really important’ (P2)* |
| **Support** | Daily activities | *`My children do all the groceries, I don’t do anything’’ (P17)* |
|  | Hypervigilance informal caregiver | *`They tell be to be careful the all the time’ (P17)* |
|  |  | *`If I do too much and I get complaints, my husband becomes angry and tells me to sit down.’ (P10)* |
|  |  | *`My husband does all the groceries and cooking and tells me to relax’ (P7)* |
|  | Cardiac rehabilitation | *`It would be great if there would be someone next to you all the time to make an ECG and tell you nothing is wrong’ (P2)* |
|  |  | *`Certain things I would like to have re-confirmed’ (P7)* |
|  |  | *`I want to participate in cardiac rehabilitation to gain confidence so that afterwards I can start exercising alone’ (P5)* |
|  |  | *`I would feel anxious if I started exercising without guidance. It’s about confidence. I can do it, but it would not feel right’ (P16)* |
|  |  | *`I hope that I will benefit from cardiac rehabilitation and that afterwards I will be able to take the bike instead of the car to do my groceries’ (P5)* |
