# Supplementary material for: Design of a Remote Coaching Program to Bridge the Gap From Hospital Discharge to Cardiac Rehabilitation: Intervention Mapping Study
Source: JMIR Cardio. 2022 May 25;6(1):e34974. doi: 10.2196/34974 (PMC9178457; doi:10.2196/34974)
Supplement: Multimedia Appendix 4 [file cardio_v6i1e34974_app4.docx]

Multimedia Appendix 4. Determinant matrix.

| **Performance objective** | **Theoretical determinants** | | | | |
| --- | --- | --- | --- | --- | --- |
|  | **Knowledge** | **Skills** | **Attitudes** | **Social influence** | **Self-efficacy** |
| *Content of eHealth intervention* | | | | | |
|  | | | | | |
| *Patients gain knowledge on how CAD and revascularization affects their bodies and health.* | Understand what happened to their heart and how this affects their body. | Possess the skill to deal with post-operative complaints. | Accept that they had a cardiac procedure and have post-procedural complaints as a consequence. | Informal caregivers support patients in dealing with post-procedural pain. | Patients feel confident that they are capable of dealing with post-procedural complaints. |
| *Patients gain knowledge about medication and side effects.* | Understand the function of medication and know about possible side effects and understand the necessity of adhering to their medical treatment plan. | Adhere to medication, recognize possible side effects and consult a health care provider if necessary. | Acknowledge that medication aids the recovery process.  Actively monitor medication treatment plan and (side) effects. | Informal caregivers support patients with adhering to their medical treatment plan and help dealing with possible side effects. | Feel confident about adhering to their medical treatment plan. |
| *Patients know which daily physical activities they can and can’t do safely after hospital discharge and are physically active.* | Understand the necessity of physical activity and understand how their cardiac procedure influences their ability to perform daily physical activities | Gradually build up physical activities and develop alternative strategies to perform daily physical activities. | Having a positive outlook on daily physical activity. | Informal caregivers support patients in building up physical activities and stimulate alternative activities if necessary. | Feeling confident about doing daily physical activities and gradually expanding the repertoire of activities. |
| *Patients can deal with the psychosocial consequences of CAD* | Having knowledge about the impact of psychological distress on their body and understanding the difference between harmful and harmless body signals. | Being able to effectively communicate about psychological distress and are able to distinguish harmful and harmless body signals. | Acknowledge that they suffer from psychological distress and have distressing body signals. | Informal caregivers support patients suffering from psychological distress after hospital discharge | Effectively seeking (professional) support if needed. |
| *Health care providers assess patients and informal caregivers needs in the first week after hospital discharge.* | Have knowledge about common physical and psychological problems of patients and informal caregivers in various subgroups. | uses assessment skills to assess needs of patients and informal caregivers. | Acknowledges that a broad and thorough assessment of patients and informal caregivers needs is essential. | Effectively assess the social environment of the patient and involve the informal caregivers during the assessment. | Feels confident and capable in performing a comprehensive needs assessment. |
| *Health care providers give tailored information and support to cardiac patients and their informal caregivers* | Have comprehensive knowledge about needs of cardiac patients and patients. | Are able to tailor the information to subgroups of patients. | Offers remote supports to patients and informal caregivers in the first phase after hospital discharge. | Coaches patient in mobilizing relevant social support. | Has confidence in coaching patients using an eHealth platform. |

* In this table, *patients* also refers to *informal caregivers*
