# Supplementary material for: Design of a Remote Coaching Program to Bridge the Gap From Hospital Discharge to Cardiac Rehabilitation: Intervention Mapping Study
Source: JMIR Cardio. 2022 May 25;6(1):e34974. doi: 10.2196/34974 (PMC9178457; doi:10.2196/34974)
Supplement: Multimedia Appendix 5 [file cardio_v6i1e34974_app5.docx]

Multimedia Appendix 5. Program strategy.

| **Theoretical determinants** | **Theory based methods** | **Practical strategies** |
| --- | --- | --- |
| *Patient and informal caregiver* | | |
|  | | |
| *Knowledge*   - *Understanding* coronary artery disease, procedures (PCI/CABG), function of medication and side effects, necessity of physical activity and influence of coronary artery disease on daily activities, origin of psychological distress and body signals. | Belief selection,  Tailoring,  individualization.  Persuasive communication,  Using imagery,  Elaboration | *Consult with a health care provider.*   - Assessment of beliefs, encouraging positive beliefs, weakening negative beliefs, introducing new beliefs. Information is tailored for subgroups and individualized. - Video clips with consistent information about medication use, coronary artery disease, procedures (PCI/CABG), physical activities and psychological distress. The content of videoclips is evaluated with the health care provider during the next consultation. |
| *Skills*   - Dealing with postprocedural pain,  adhering to medical treatment plans and dealing with side effects. - Gradually building up physical activity. - Dealing with psychological distress and discriminating between harmful and harmless body signals. | Goal setting  Reattribution training  Self-monitoring of behavior  Improving physical and emotional states,  Setting graded tasks. | *Consult with a health care provider.*   - Patients and health providers formulate concrete goals for the first phase after hospital discharge - Patients study video clips about dealing with post-procedural pain, adhering to medical treatment plan and dealing with side effects, building up physical activity, dealing with psychological distress and body signals. The content of videoclips is evaluated with the health care provider during the next consultation. |
| *Attitude*   - Accepting current status, actively monitor medication treatment plan, having a positive outlook on daily physical activity. Acceptance of psychological distress. | Shifting perspective, Elaboration,  Direct experience,  Repeated exposure  Motivational interviewing | *Consult with a health care provider.*   - During consultation the patient is being coached in looking at their situation from a different perspective (health problems, psychological distress and physical activity). - Patient learns to challenge him- or herself and discover the growing possibilities despite boundaries. - Patient studies video clips to shift perspective, perform exercises and evaluates with health care providers. |
| *Social influence*   - Informal caregiver supports patient in dealing with post-procedural pain, adhering to treatment plan, building up physical activities, dealing with psychological distress and distressing body signals. - Patients support each other by sharing experiences with each other. | Prompting hiding of the unpopular behavior or shifting attention away from the behavior.  Mobilizing social support  Providing opportunities for social comparison | Consult with a health care provider.   - During consults with health care providers the informal caregiver is actively involved and coached by health care providers about supporting patients. - Patients without a partner are coached on how to reach out and find social support through eHealth portal. - Discussion board where patients (and informal caregivers) share experiences. Are brought into contact with each other. |
| *Self-efficacy*   - Confident about dealing with postprocedural pain, adhering to medical treatment plan, physical activity, psychological distress and body signals. | Motivational interviewing  Guided practice  Re-attribution  Self-monitoring  Goal setting  Planning coping responses | *Consult with a health care provider.*   - Self-efficacy of patients is stimulated by changing beliefs, attitudes and experiences. Patient formulates concrete, feasible goals and reflects on progress in the eHealth environment. |
| ***Health Care providers*** | | |
| *Knowledge*   - *Having* knowledge about common physical and psychological problems of patients and informal caregivers after hospital discharge. - Having knowledge about needs of cardiac patients and patients. | Chunking  Advance organizers  Discussion | - Health care providers follow mandatory workshops and gain knowledge about typical information and support needs of cardiac patients and know the best didactical approaches for assessing needs, using shared decision making. Health care providers gain knowledge about supporting and coaching cardiac patients. |
| *Skills*   - Using assessment skills to assess needs of patients and informal caregivers - Are able to tailor the information to subgroups of patients. | Guided practice  Feedback  Tailoring | - Health care providers follow mandatory workshops aimed at learning and practicing assessment skills together with peers. - Learning to communicate with various subgroups of patients. |
| *Attitudes*   - Acknowledges that a broad and thorough assessment of patients and informal caregivers needs is essential. - Offers remote supports to patients and informal caregivers in the first phase after hospital discharge | Shifting perspective,  Direct experience,  Repeated exposure | - Health care providers are aware of the necessity of a thorough assessment to effectively start remote coaching. Health care providers are positive about remote coaching and using the eHealth platform. |
| *Social Influence*   - Effectively assesses the social environment of the patient and involves the informal caregiver during the assessment - Coaches patient in mobilizing social support | Mobilizing social support  Providing opportunities for social comparison | - Health care providers actively assesses barriers and facilitators for the intervention in the social environment of the patients. - The health care provider coaches the patients in gaining social support (informal caregiver, hospital, cardiac rehabilitation center). |
| *Self-efficacy*   - Feels confident and capable in performing a comprehensive needs assessment - Has confidence in coaching patients using an eHealth platform | Guided practice  Self-monitoring  Goal setting | - Health care providers actively practices assessment skills with peers and gains trust in performing assessment. - Health care provider actively practices coaching skills with peers using the eHealth platform |
