# Supplementary figures and images for: Design of a Remote Coaching Program to Bridge the Gap From Hospital Discharge to Cardiac Rehabilitation: Intervention Mapping Study
Source: JMIR Cardio. 2022 May 25;6(1):e34974. doi: 10.2196/34974 (PMC9178457; doi:10.2196/34974)

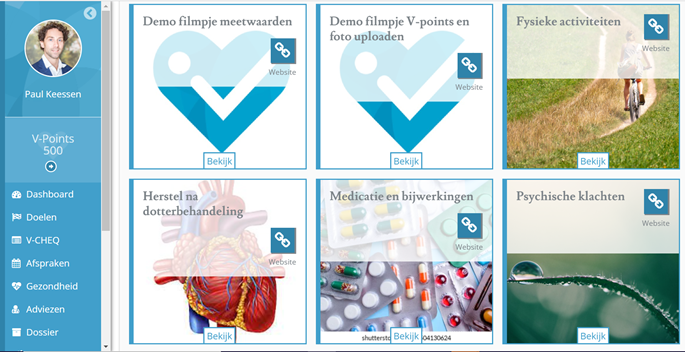

Supplement: Multimedia Appendix 6 [file cardio_v6i1e34974_app6.png]

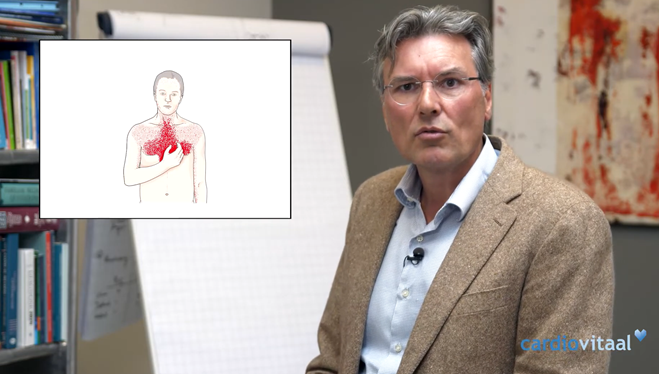

Supplement: Multimedia Appendix 7 [file cardio_v6i1e34974_app7.png]

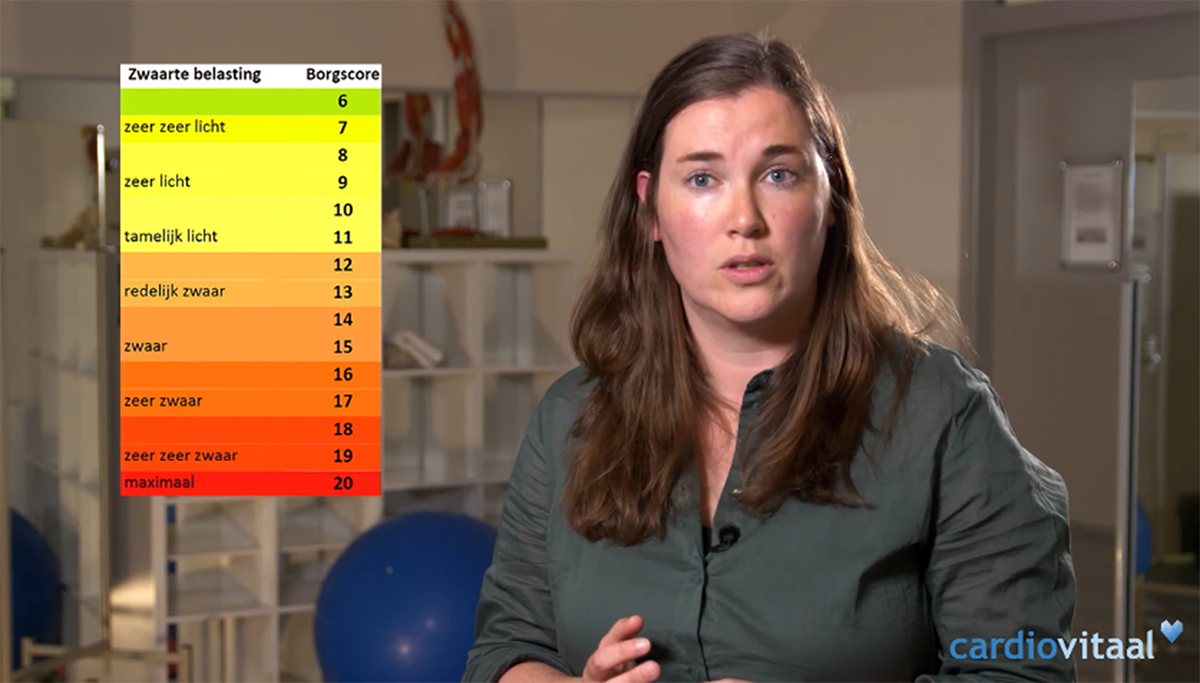

Supplement: Multimedia Appendix 8 [file cardio_v6i1e34974_app8.png]
